# Chlormethine gel in combination with other therapies for treatment of mycosis fungoides: a review with patient cases

**DOI:** 10.3389/fmed.2023.1308491

**Published:** 2024-01-11

**Authors:** Marco Ardigò, Neda Nikbakht, Miriam Teoli, Laura Gleason, Liliana Crisan, Christiane Querfeld

**Affiliations:** ^1^Clinical Dermatology Department, San Gallicano Dermatological Institute, IRCCS, Rome, Italy; ^2^Department of Biomedical Sciences, Humanitas University, Pieve Emanuele, Italy; ^3^Department of Dermatology and Cutaneous Biology, Thomas Jefferson University, Philadelphia, PA, United States; ^4^Division of Dermatology and Department of Pathology, City of Hope National Medical Center and Beckman Research Institute, Duarte, CA, United States

**Keywords:** mycosis fungoides, chlormethine gel, combination therapy, phototherapy, retinoids, mogamulizumab

## Abstract

Topical chlormethine gel has been approved as monotherapy for treatment of adult patients with mycosis fungoides (MF), the most common form of cutaneous T-cell lymphoma. In clinical practice, chlormethine gel is often combined with other skin-directed or systemic therapies to optimize response and target recalcitrant lesions. Positive outcomes with combination regimens using chlormethine gel and topical corticosteroids, phototherapy, retinoids, methotrexate, or interferon-α have been reported in literature. However, there are no treatment guidelines on the use of combination regimens with chlormethine gel. To provide real-world evidence and guidance on the use of chlormethine gel combination regimens, several cases of patients treated with chlormethine gel combined with phototherapy (*n* = 5), retinoids (*n* = 16), or mogamulizumab (*n* = 3) are presented. These different combination regimens showed promising results. Most patients had a complete or partial response following treatment and the combinations were well-tolerated over extended treatment periods. Patients receiving chlormethine gel with retinoids had long-term periods of remission, even after treatment discontinuation. Durations of response of up to 3 years were observed in these patients. This long-term disease control may be the result of disease-modifying effects of chlormethine. Previous studies have shown targeted reductions in malignant T-cell clones in patients treated with chlormethine gel as well as improved post-treatment responses. Further research is needed to determine the effectiveness and safety of combination treatment regimens with chlormethine gel and to assess the impact chlormethine gel has on disease control.

## Introduction

### Mycosis fungoides

Patients with mycosis fungoides (MF), the most common form of cutaneous T-cell lymphoma, often present with patches or plaques on the skin (1, 2). While MF tends to follow a slow, indolent course during early stages of disease, patients may develop tumors, erythroderma, and blood or organ involvement when the disease progresses to more-advanced stages (3). Patients with early-stage MF generally have a favorable prognosis, but this worsens with disease progression (4). Even during early-stage disease, patients can suffer from severely reduced quality of life due to symptoms such as insomnia, anxiety, and pain (5, 6). In addition, many patients experience itching that can be severe and debilitating (7).

Treatment choice for MF depends on disease stage and is aimed at reducing symptoms, preventing disease progression, and improving quality of life (1, 3, 8, 9). For treatment of early-stage MF, skin-directed therapies such as phototherapy, topical corticosteroids, chlormethine, or retinoids are recommended. Topical chlormethine is currently recommended as a first-line treatment option for stage IA–IIA MF by multiple treatment guidelines (1, 3, 8, 9). For patients with more-advanced disease, the use of systemic agents is recommended. The addition of skin-directed therapies during advanced-stage disease may help alleviate symptoms and reduce the time to response compared with systemic therapies alone.

### Chlormethine gel

The bifunctional alkylating agent chlormethine can inhibit rapidly proliferating cells by binding to and crosslinking DNA (10). Early chlormethine preparations were aqueous or compounded ointment-based formulations that could be challenging for patients to use. The chlormethine 0.016% w/w topical gel formulation (equivalent to 0.02% CL HCl) was specifically developed for treatment of patients with MF. The gel formulation is non-greasy and quick drying, which makes it easy to apply for patients or caregivers at home and can help encourage compliance. It has been approved in the US, the EU, and other countries worldwide (11–13). The pivotal phase 2 trial compared chlormethine gel monotherapy with chlormethine ointment monotherapy (14). The use of concomitant therapies, including topical or systemic corticosteroids, was not allowed during the trial. The Composite Assessment of Index Lesion Severity (CAILS) response rate, the primary efficacy endpoint of the trial, was 58.5% for patients treated with chlormethine gel. This was non-inferior to the response rate seen with chlormethine ointment (47.7%). No drug-related serious adverse events (AEs) were observed during the study.

In clinical practice, chlormethine gel is often combined with other skin-directed or systemic therapies to achieve the best possible outcomes for patients. Using systemic therapies in combination with chlormethine gel is unlikely to result in any drug-drug interaction effects, as pharmacokinetic analysis has shown that chlormethine gel is not systemically absorbed (15). Permeation studies with chlormethine gel showed that the gel delivered more chlormethine with a higher rate to epidermal membrane compared with dermatomed skin (16); this suggests that minimal amounts of chlormethine pass through epidermal tissue to reach dermal tissue, and correlates with the data on lack of systemic absorption (15). Further investigation of the mode of action of chlormethine gel showed that it induces DNA double-stranded breaks as well as expression of proapoptotic *CASP3*, mainly in malignant MF skin T cells. Use of chlormethine gel also decreased the expression of genes that are involved in alkylated nucleotide excision (17). These results suggest that chlormethine gel may have synergistic effects, especially when combined with other skin-directed therapies.

Data on the use of different combination therapies with chlormethine gel in clinical practice are scarce. A recent expert consensus concluded that the use of combination therapies with chlormethine gel, especially in later stages of disease, should be decided by clinicians on an individual basis, given the lack of evidence (18). Herein, we review the available data from literature on combination regimens with chlormethine gel and present several cases of patients who received chlormethine gel combination regimens.

## Chlormethine gel combination regimens

The largest real-world study to date that investigated chlormethine gel was the PROVe study. During this study, 298 patients with MF who were treated with chlormethine gel were monitored for up to 2 years (19). Combination treatment regimens were common in the PROVe study; 78% of patients used other skin-directed therapies and 30% used systemic therapies in combination with chlormethine gel during the study period. The effectiveness of chlormethine gel monotherapy could not be assessed due to the low number of patients receiving this treatment. The three most common combination treatments were topical corticosteroids (60%), phototherapy (21%), and oral bexarotene (16%). Response rates in patient groups receiving different concomitant therapies were similar. A *post-hoc* analysis of the PROVe data showed that most concomitant therapies used with chlormethine gel were initiated prior to the start of chlormethine gel. In over half of cases the concomitant therapy was used for at least 12 months (20).

The combination of chlormethine gel with topical corticosteroids has been seen in other real-world studies as well, and this appears to be a relatively common strategy (21–24). Topical corticosteroids are also used in clinical practice to reduce the risk for contact dermatitis, which is one of the most common AEs associated with chlormethine gel treatment (14, 25). A prospective, randomized, controlled study directly compared treatment with chlormethine gel alone to the combination of chlormethine gel with the topical corticosteroid triamcinolone (26). The addition of triamcinolone reduced the occurrence of contact dermatitis in lesions treated with combination treatment compared with chlormethine gel alone. Increased CAILS improvements were also observed in lesions treated with combination therapy; however, this was not statistically significant when compared with chlormethine gel monotherapy.

The use of combination regimens with chlormethine gel and systemic therapies has also been reported in literature. In a study investigating real-world efficacy and safety of chlormethine gel, 11 of 23 enrolled patients received chlormethine gel in combination with either methotrexate or pegylated interferon (IFN)α-2A (27). Clinical responses were seen when chlormethine gel was added to treat localized tumor lesions that were refractory to systemic treatment in 5 patients with stage IIB MF. One patient achieved a complete response (CR) at month 9 of combination treatment with chlormethine gel and pegylated IFNα-2A.

Taken together, these data indicate that chlormethine gel is regularly used in combination with other therapies, including skin-directed and systemic agents, with positive outcomes. Currently, there are no recommendations regarding combining chlormethine gel with other therapies and no clear treatment patterns are seen in real-world studies. This can make it difficult for clinicians when deciding if they can combine the gel with other therapies, and whether to continue or discontinue other treatments when prescribing chlormethine gel.

## Patients treated with chlormethine gel combination regimens

To provide real-world evidence and guidance on the use of chlormethine gel combination regimens, we present several cases of patients with MF who were treated with chlormethine gel combined with phototherapy, retinoids, or mogamulizumab.

### Chlormethine gel with phototherapy

Phototherapy, most commonly narrowband ultraviolet B (nbUVB), is a first-line treatment option often used during early-stage MF (28). Response rates seen with nbUVB treatment range from 54 to 91%. As reported in the PROVe study, phototherapy is regularly combined with chlormethine gel in clinical practice (19). Here we present results from a retrospective chart review of 5 patients who were treated simultaneously with chlormethine gel and nbUVB (Table 1). Three patients received combination therapy for at least 6 months and are discussed in more detail.

**Table 1:** Patients treated with CL gel-containing combination treatment regimens.

	**CL gel + phototherapy^a^**	**CL gel + retinoids**	**CL gel + mogamulizumab**
	***n* = 5**	***n* = 15**	***n* = 3**
**Age, range**	54–71	48–70	57–83
**Sex**, ***n*** **(%)**			
Male	4 (80)	8 (53)	1 (33)
Female	1 (20)	7 (47)	2 (67)
**MF stage**, ***n*** **(%)**			
IA	1 (20)	0	0
IB	3 (60)	1 (7)	0
IIA	0	10 (67)	0
IIB	1 (20)	4 (27)	0
IIIB	0	0	1 (33)
IVA1	0	0	1 (33)
IVA2	0	0	1 (33)
**Treatment combined with CL gel, n (%)**			
nbUVB	5 (100)	0	0
Acitretin	0	8 (53)	0
Bexarotene	0	7 (47)	0
Mogamulizumab	0	0	3 (100)
**Months of combination therapy, range**	2–14	4–11	6–8
**CL gel treatment schedule, n (%)**			
Once daily	0	15 (100)	1 (33)
2–4 times per week	4 (80)	0	2 (67)
Once weekly	1 (20)	0	0
**Skin-related adverse events, n (%)**			
Yes	3 (60)	12 (80)	2 (67)
No	2 (40)	3 (20)	1 (33)
**Adverse event management, n (%)**			
Topical steroids	0	6 (40)	0
Decreased CL gel schedule	0	6 (40)	0
Discontinuation	1 (20)	0	1 (33)
**Best response**, ***n*** **(%)**			
CR	1 (20)	10 (67)	2 (67)
PR	3 (60)	5 (33)	1 (33)
SD	1 (20)	0	0
PD	0	0	0

^a^One patient reinitiated chlormethine gel + nbUVB after relapse; only the first combination treatment period is included in the table.

CL, chlormethine; CR, complete response; nbUVB, narrowband ultraviolet B; PD, progressive disease; PR, partial response; SD, stable disease.

The first patient was a 62-year-old man with stage IB MF. The patient had previously received total skin electron beam therapy (TSEBT), chlormethine ointment, clobetasol ointment, and psoralen and ultraviolet A. Chlormethine gel treatment was initiated at a frequency of 3–4 times per week when the patient had a modified Severity-Weighted Assessment Tool (mSWAT) score of 50. After 6 months of treatment, the patient achieved a partial response (PR) with an mSWAT score of 25. At that time, nbUVB was added to the treatment regimen at a frequency of 2–3 times per week. Chlormethine gel and nbUVB were given on different days. Over the next 8 months, the mSWAT score reduced to 11.5. The only AEs experienced by the patient were dryness and itchiness of the skin. Three months later, the mSWAT score had increased to 22 and the patient discontinued chlormethine gel and nbUVB combination therapy to enroll in a clinical trial.

**Figure 1:**
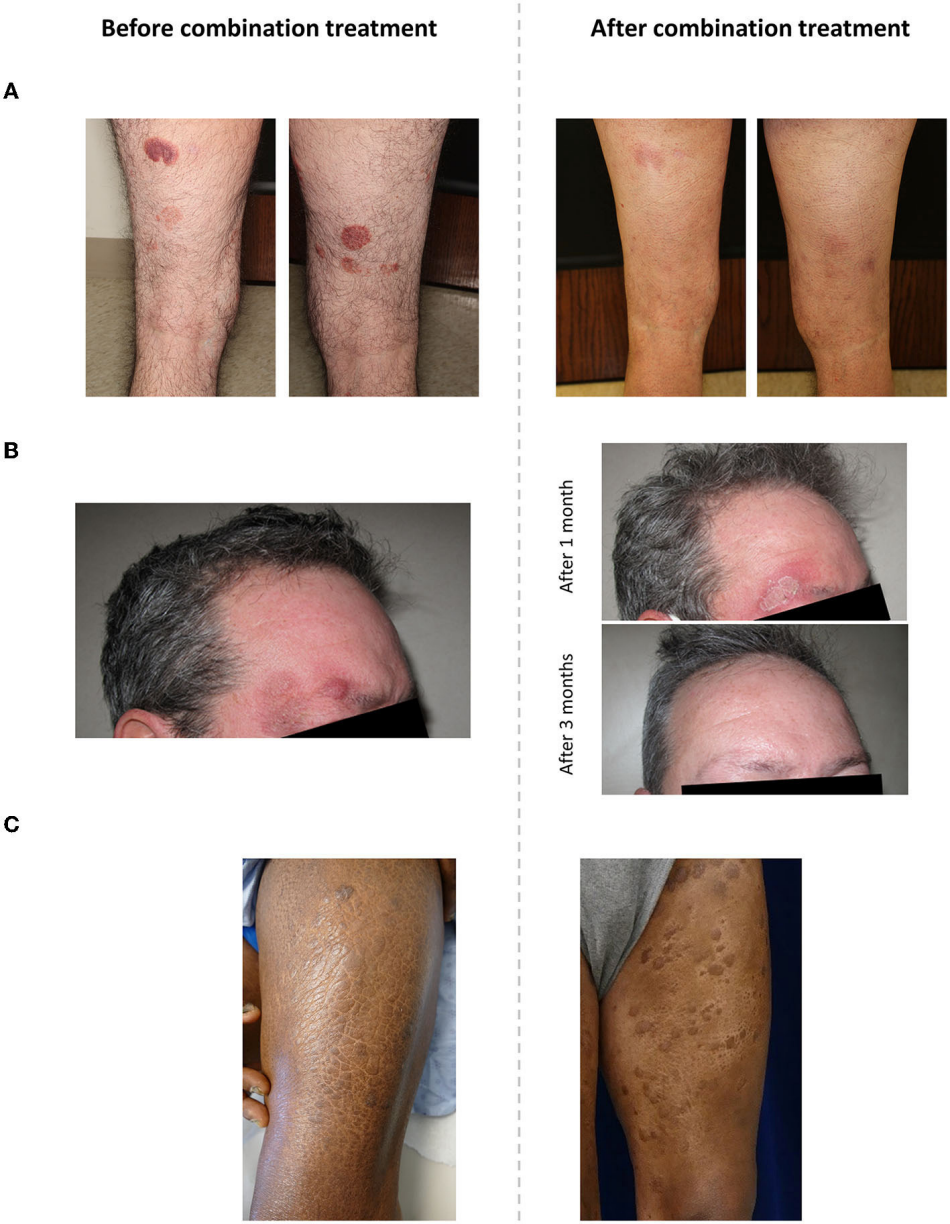
Representative images of patients receiving combination therapy with chlormethine gel and **(A)** narrowband ultraviolet B, **(B)** interferon-α and acitretin, and **(C)** mogamulizumab.

The second case was a 58-year-old man with stage IA MF who had previously received chlormethine gel monotherapy, pimecrolimus, and clobetasol. Chlormethine gel treatment was initiated at a frequency of 4 times per week when the patient had an mSWAT score of 5. One month after initiating chlormethine gel, the mSWAT score had increased to 9. Another month later, nbUVB was added to the treatment regimen, 2–3 times per week. Eight months after the addition of nbUVB, the patient achieved a CR and combination treatment was discontinued (Figure 1A). No AEs were experienced with combination therapy. The CR was maintained for 3.5 months, after which an mSWAT of 0.5 was observed and the patient started bexarotene treatment. Three years later, the patient had progressive disease with an mSWAT of 2 and chlormethine gel with nbUVB combination therapy was reinitiated. An initial mild decrease in mSWAT was observed after treatment reinitiation; however, 8 months later the mSWAT score increased to 14. The patient discontinued combination therapy and began treatment with brentuximab vedotin and localized radiation at the time of analysis.

The third patient was a 64-year-old man with stage IIB MF who was heavily pretreated. He had previously received localized radiation, TSEBT, bexarotene, brentuximab vedotin, nbUVB monotherapy, tacrolimus, pimecrolimus, chlormethine gel monotherapy, and imiquimod. Chlormethine gel treatment was initiated at a frequency of every other day when the patient had an mSWAT score of 4.5. At this time the total percentage body surface area (BSA) was 2.5%, consisting of 1.5% patch, 0.5% plaque, and 0.5% tumor. Four months later, the mSWAT score had increased to 9.5. At this time, nbUVB was added to the treatment regimen at a frequency of 2–3 times per week. After 4 months of combination therapy, the patient had stable disease with a modest decrease in mSWAT to 7. During combination therapy, the patient experienced a rash with chlormethine gel but was able to remain on treatment. Three months later, the patient experienced progressive disease with an mSWAT score of 17 and he enrolled in a clinical trial.

### Chlormethine gel with retinoids

Topical and systemic retinoids have been an important part of the treatment armamentarium for patients with MF for years (29), and oral retinoids, such as bexarotene, are often used as the first systemic therapy for patients (30). Systemic retinoids have also been combined with other MF treatments and were shown to be well-tolerated and associated with good response rates in small studies (29, 31). After topical corticosteroids and phototherapy, oral bexarotene was the third most common combination therapy seen in the PROVe study (19). Here we present results from a retrospective chart review of 15 patients who received combination therapy with chlormethine gel and bexarotene or acitretin (Table 1). Included patients had stage IB–IIB MF, including 10 cases of stage IIA MF that are rarely seen in clinical practice, and previously received phototherapy (*n* = 10), IFN (*n* = 3), or photopheresis (*n* = 2). Two patients received combination therapy for at least 6 months and are discussed in more detail. In addition, a challenging case of a patient who received chlormethine gel, acitretin, and IFNα combination therapy is presented.

The first patient was a 48-year-old man with stage IIA MF who had previously been treated with photopheresis. Oral acitretin treatment was initiated at a dose of 10 mg per day and after 6 months of treatment, the patient's disease burden had increased to a BSA of 40% and an mSWAT score of 125. At this time, chlormethine gel was added to the treatment regimen at a once-daily frequency. The patient had irritant contact dermatitis while receiving combination therapy, which was managed through the addition of topical corticosteroids. He received combination treatment for a total of 7 months and then discontinued due to a CR. At the time of analysis, the patient remained in remission with a duration of response of 2 years.

The second patient was a 63-year-old man with stage IIA disease who had previously received phototherapy. Oral acitretin was initiated at a daily dose of 10 mg. Two months later, when the patient had a BSA of 40% and mSWAT score of 135, chlormethine gel was added to the treatment regimen. The gel was applied once daily and tolerated without AEs. The patient achieved a CR after 11 months of combination therapy and treatment was discontinued. At the time of analysis, the CR was maintained with a duration of response of 3 years.

Overall, all 15 patients receiving chlormethine gel in combination with bexarotene or acitretin responded very well to treatment (Table 1). Ten patients had a CR after combination treatment and the remaining 5 patients had a PR. The median duration of response was 2 years, with a range of 1–3 years. All patients also discontinued combination treatment in response to remission and they did not require maintenance therapy to maintain their response.

One additional challenging case outside the retrospective review of 15 patients was studied. This patient was a 48-year-old man with stage IIB MF. He had been receiving treatment with 3,000,000 units of IFNα per week plus daily 25 mg oral acitretin for 8 months when he developed a persistent and progressively enlarging nodule on the right side of his forehead. When the nodule had been apparent for ~4 weeks, the patient initiated daily chlormethine gel treatment while continuing to receive IFNα and acitretin. Chlormethine gel was chosen as an alternative option to using local radiotherapy for the nodule, as the patient did not wish to receive radiotherapy. After a month of treatment, the patient experienced edema and crusty patches that was identified as contact dermatitis. Following a discussion, the patient agreed to proceed with chlormethine gel treatment. Six months after combination treatment initiation, the patient achieved a CR with complete disappearance of the nodule, and chlormethine gel was discontinued (Figure 1B). Subsequently, the contact dermatitis resolved within 2 weeks. At the time of analysis, the patient was receiving bexarotene treatment and the CR for the nodular lesion was maintained.

### Chlormethine gel with mogamulizumab

Mogamulizumab is a first-in-class monoclonal antibody that binds to C-C chemokine receptor 4 (CCR4), which is expressed on the surface of tumor cells in T-cell malignancies (32). An open-label, phase 3, randomized, controlled trial compared mogamulizumab and vorinostat in patients with relapsed/refractory MF or Sézary syndrome. Longer median progression-free survival was seen in patients treated with mogamulizumab (7.7 months) compared with vorinostat (3.1 months) (32). Mogamulizumab has a favorable safety profile (33) and it functions through antibody-dependent cellular cytotoxicity, without inducing complement-dependent cytotoxicity. The target of mogamulizumab, CCR4, is only expressed on T-helper 2 cells (malignant cells) and regulatory T cells. As a result, no cytotoxic effects are seen in the skin, making mogamulizumab ideal to combine with other therapies. A recent study demonstrated that the combination of bexarotene and mogamulizumab resulted in responses in four patients with advanced disease after the failure of bexarotene alone (33). Here we present results from a retrospective chart review of three patients with advanced-stage MF who received combination therapy with chlormethine gel and mogamulizumab (Table 1).

The first case was a 57-year-old woman with stage IVA1 MF who was previously treated with extracorporeal photopheresis (ECP) and nbUVB, IFNα, and brentuximab. Mogamulizumab was initiated as per standard regimen once weekly for the 1st month and then once every 2 weeks, alternating weekly with ECP for ~20 months. Treatment was discontinued when the patient achieved a CR. Six months later, the patient had developed new plaques (BSA of 11%; mSWAT of 18) with a low circulating Sézary cell count of ~6%, resulting in the decision to restart mogamulizumab and add chlormethine gel to the treatment regimen. Chlormethine gel was applied 3 times per week. No AEs were reported during combination therapy. The patient achieved CR after 7 months of combination treatment with no abnormal circulating T cells. However, the patient developed mogamulizumab-associated rash (MAR) affecting 4% of BSA that was confirmed by skin biopsy; recurrent cutaneous T-cell lymphoma was not seen. At the time of analysis, the patient was still being treated with chlormethine gel tapered to once monthly; mogamulizumab was discontinued.

The second patient was an 83-year-old woman with stage IIIB MF. Previous treatment consisted of topical corticosteroids and mogamulizumab. Treatment with mogamulizumab was initially held due to clinical remission but restarted 4 months later, as the patient developed new erythematous plaques, consistent with MF per histopathologic evaluation. A few weeks later, chlormethine gel was added to the treatment regimen at a frequency of 3 times per week. At the time of initiating the combination regimen, the patient had a BSA of 20% and mSWAT score of 35. The patient had mild itching and MAR but was able to continue treatment without treatment adjustments. After 6 months of treatment, the patient discontinued combination therapy after achieving a CR.

The third patient was a 60-year-old man with stage IVA2 MF who had been heavily pretreated. Prior therapies he received were bexarotene, IFNα, topical steroids, ECP, and vorinostat. Treatment with mogamulizumab was initiated at a frequency of once weekly for 1 month and then continued at a frequency of once every 2 weeks. Ten months later, daily chlormethine gel treatment was added to the regimen. The patient responded to treatment with a PR but experienced itching and MAR. Mogamulizumab was intermittently held, but eventually the patient had to discontinue combination therapy after 7 months due to the MAR. While it was difficult to separate the MAR from MF, it did appear that the MF plaques were cleared or improved after chlormethine gel treatment (Figure 1C). At the time of analysis, the patient had a BSA of 35% and mSWAT score of 70, attributed to MAR.

## Summary and discussion

Chlormethine gel is an approved skin-directed therapy for patients with MF that is often combined with other therapies in clinical practice (19). Chlormethine gel can be combined with different types of other skin-directed therapies or with systemic therapies to optimize response and target recalcitrant lesions. For patients receiving systemic therapy, the addition of chlormethine gel for treatment of new plaques that are resistant to the current treatment (which has proven beneficial for the patient) may be preferable to initiating a different treatment regimen, as patients may not respond as well to this. Combination regimens of chlormethine gel and topical corticosteroids, phototherapy, retinoids, methotrexate, or IFNα have been reported in literature (19, 21–24, 26, 27, 34). No treatment guidelines are currently available on the use of combination regimens with chlormethine gel.

Patients presented in this review were treated with combinations of chlormethine gel and nbUVB, retinoids, or mogamulizumab. These different combination regimens showed promising results. Four out of five patients treated with chlormethine gel and nbUVB had CR or PR as best response, while one had stable disease as best response. In addition, all patients receiving chlormethine gel and retinoids or chlormethine gel and mogamulizumab had CR or PR as best response. It should be noted that given the real-world nature of the data, it is difficult to conclude whether the positive outcomes were due to one of the agents or to the combination therapy. However, combination of chlormethine gel with various agents resulted in treatment benefit for patients. More studies are needed on this topic.

One of the reasons that combination therapies with chlormethine gel are effective could be that the different mechanisms of action result in improved outcomes. Chlormethine can induce DNA double-stranded breaks as well as expression of proapoptotic factors, and mainly acts in epidermal tissue (16, 17). The mechanisms of action of nbUVB are thought to include interference with immunity by inhibition of antigen presentation by Langerhans cells and upregulation of cytokines. In addition, nbUVB may suppress proliferation of clonal T cells and induce apoptosis of atypical lymphocytes in the epidermis and papillary dermis (35, 36). The effects of retinoids are mediated through two families of intracellular receptors: retinoic acid receptors and retinoic X receptors. Retinoids can modulate cell proliferation and differentiation as well as immunoregulation of epithelial cells. In addition, they may induce cellular apoptosis and DNA fragmentation in sensitive T cells (37). Finally, mogamulizumab targets CCR4, which can facilitate T-cell migration to the skin through skin-associated chemokines. In addition to reducing circulating CCR4-positive malignant cells, mogamulizumab may also help reduce native regulatory T cells (38).

As with any treatment, the risk-benefit ratio of the combination therapies needs to be considered and, particularly in MF, the potential risk of developing secondary skin cancers is of special interest. One retrospective study showed that eight of 203 patients with MF, who received aqueous or compounded ointment-based formulations of chlormethine as initial treatment, developed cutaneous squamous or basal cell carcinoma but none were related to treatment with chlormethine as monotherapy (39). The two patients who received topical chlormethine as monotherapy (2/203, < 1.0%) experienced the secondary cancers on the face, where topical chlormethine was not applied (39). Moreover, six of the eight patients who developed secondary cancers had received multiple treatment types after chlormethine, including TSEBT or phototherapy, which have been associated with an increased risk of secondary skin cancer (40). One older study noted that two of six patients who developed malignant melanoma after TSEBT had received additional therapy with chlormethine and oral psoralen and ultraviolet A (41). Overall, there is a lack of reports of secondary cancer on sites of application using topical chlormethine monotherapy. A 30-year population-based cohort study in Denmark showed that treatment with chlormethine monotherapy is not associated with an increased risk of secondary cancers in patients with MF (42). In the pivotal randomized trial with chlormethine gel, none of the non-melanoma skin cancer cases diagnosed throughout the 24-month observation period were considered related to chlormethine (gel or ointment) use, as they occurred in patients with a history of skin cancer or who had received previous treatment with therapies recognized to increase the risk of skin cancer (14). Finally, in clinical practice, no general increase in skin cancers has been observed in patients who received chlormethine gel with phototherapy compared with phototherapy alone. However, more long-term data on possible carcinogenic effects are needed. For patients receiving combination therapy, additional considerations may need to be made, such as avoiding phototherapy for patients with very light skin or a prior history of skin cancer, and the use of offset treatment schedules to minimize risks.

There were differences in MF stage distribution between the combination regimens investigated. Most patients who received chlormethine gel and nbUVB (4/5) or retinoids (11/15) had early-stage (IA–IIA) disease, while all patients who received chlormethine gel and mogamulizumab had advanced-stage MF (IIIB–IVA). While nbUVB and retinoids are often recommended during early-stage MF (9), mogamulizumab was approved for adult patients with relapsed or refractory MF or Sézary syndrome who received at least one prior systemic therapy (43). The frequency of chlormethine gel treatment also differed between the combination groups. All patients receiving retinoids combination therapy were using chlormethine gel on a daily basis, while those receiving nbUVB or mogamulizumab combination therapy mainly applied it at lower frequencies. While the recommended treatment frequency of chlormethine gel, per the prescribing information, is once daily (11, 12), several patients in this case series received the gel at a lower frequency. This is not uncommon in real-world clinical practice with chlormethine gel. On the basis of clinical assessments, clinicians may decide to adjust the frequency of application of chlormethine gel. The main reason for using or starting chlormethine gel at a reduced frequency is to improve tolerance by reducing adverse reactions, particularly during the 1st months of therapy. With an initial reduced dosing schedule, the dose can be titrated up or down based on clinical response or AEs (24). Multiple studies have presented data from patients who received chlormethine gel at frequencies ranging from 1 to 6 times per week and good responses to treatment were observed in these studies (19, 21, 22, 25, 44). In addition, a *post-hoc* analysis of the pivotal phase 2 trial and its extension trial indicated that there was no association between the application frequency of chlormethine gel and an improved skin response per CAILS. These results imply that a reduced treatment frequency might not impact the chance of achieving a response (45). Most patients presented here did experience skin-related AEs during combination therapy, but these could often be managed by informing patients of anticipated AEs, counseling, reducing the treatment frequency, or adding topical corticosteroids to the treatment regimen. Treatment discontinuation due to AEs was rare in this collection of cases; one patient receiving nbUVB combination therapy discontinued treatment due to skin irritation and one patient receiving mogamulizumab discontinued due to MAR. MAR is the most common AE seen in patients treated with mogamulizumab, with one retrospective case series in cutaneous T-cell lymphoma observing an incidence of 68% (46). Chlormethine gel is unlikely to contribute to the risk of MAR. MAR lesions were observed individually of chlormethine-treated lesions, and chlormethine is not systemically absorbed after topical application (15). In addition, the occurrence of MAR has been linked to the depletion of regulatory T-cells caused by mogamulizumab treatment, and chlormethine gel is not known to affect regulatory T-cells (46, 47).

Many patients received combination therapy for an extended period of time, with longest treatment durations in individual patients of 14 months for nbUVB, 11 months for retinoids, and 8 months for mogamulizumab. Longer-duration treatment may be needed with chlormethine gel monotherapy or combination regimens before the best possible response is reached. Response rates with chlormethine gel monotherapy were seen to rise over time in a *post-hoc* analysis of the pivotal trial data, with a peak of response after 10 months of treatment (48). Similarly, in the PROVe study the peak response occurred at 18 months for patients with stage IA–IB disease who received chlormethine gel combination regimens (19).

Long-term periods of remission were observed in patients in this study, in particular in those receiving combination therapy with retinoids. Patients receiving this combination had durations of response of up to 3 years. Most of these patients experienced dermatitis during treatment. This long-term disease control may in part be caused by a unique effect that chlormethine appears to have on the disease evolution of MF (49). In the MIDAS study, patients were treated for 4 months, but CAILS improvements were still present after 5 and 12 months despite treatment discontinuation (26). This study also assessed the molecular identity of T-cell clones pre- and post-treatment and found that 3 individual malignant clones that were identified at baseline were significantly diminished at month 5 in a representative patient. In addition, no expansion of baseline malignant clones was seen during dermatitis flares in any patient with contact dermatitis. These targeted reductions in malignant T-cell clones, also described by Chang et al. (17), alongside clinical response appear to reflect an impact of chlormethine on the underlying pathophysiology in patients with early-stage MF. Such disease-modifying effects have also been seen with nbUVB as first-line therapy in early-stage MF (50).

## Conclusion

In conclusion, published literature and our presented cases show that different chlormethine gel-containing combination treatment regimens appear to be well-tolerated and effective. However, the available evidence is very limited and further research is needed. In addition, more evidence needs to be collected on whether the use of chlormethine gel, alone or in combination, as a first-line treatment option might lead to earlier and longer control of MF, considering the potential impact chlormethine gel may have on disease control.

## Ethics statement

Written patient consent for publication of the images was obtained using institutional patient consent forms.

## Author contributions

MA: Conceptualization, Writing – original draft, Writing – review & editing. NN: Conceptualization, Writing – original draft, Writing – review & editing. MT: Conceptualization, Writing – original draft, Writing – review & editing. LG: Conceptualization, Writing – original draft, Writing – review & editing. LC: Conceptualization, Writing – original draft, Writing – review & editing. CQ: Conceptualization, Writing – original draft, Writing – review & editing.
